# Alternative Strategies of Home Therapy in Clinical Orthopaedics during COVID-19 Pandemic: a Hand Surgeon Perspective

**DOI:** 10.5704/MOJ.2007.006

**Published:** 2020-07

**Authors:** V Cilli

**Affiliations:** Hand Surgery, Chirec Site Delta, Brussels, Belgium

Dear Editor,

I have read with interest the article by Passiatore *et al.* “The Use of Alfa-Lipoic Acid-R (ALA-R) in Patients with Mild-Moderate Carpal Tunnel Syndrome: A Randomised Controlled Open Label Prospective Study”, published in the last issue of Malaysian Orthopaedic Journal^1^.

Due to the COVID-19 pandemic, as an hand surgeon, I have to deal with a lockdown of both elective surgery and outpatient.

Due to legal restriction, in many countries taking charge of elective patients is prohibited or strictly limited, with the fear that many patients feel if requested to come to hospitals, and to the necessity to avoid, as much as possible, non-essential displacements due to the COVID-19 pandemic^2^.

I have recently reassessed conservative strategies to control or treat musculoskeletal diseases at home, or even just to manage the related-to pain. The above mentioned article focuses on the management of pathologies like carpal tunnel syndrome^1^. According to my personal experience, ALA-R appears as a very useful tool for management of nervous compression related pain and peripheral neuralgia. Nevertheless, there are many others medications that could be useful in handling typical hand surgery elective pathologies, when surgery has to be delayed or is contraindicated for patient-related reasons^3, 4^. What should be taken into account, is that different molecules are effective on different aspects of pain and disability. As an example, the Ultra Micronized Palmitoylethanolamide was found to improve the sleep-awake rhythm in these patients, thus giving the way to an improvement in pain perception and overall health conditions^5^. Other molecules are Group B vitamines and N-Acetyl Carnitine seem to be effectives on neuropathic pain , while Symptomatic slow-acting drugs for osteoarthritis (SYSADOAs), such as glucosamine and chondroitin sulfate, act on nociceptive pain, that is especially related to osteoarthritis^6^.

As a conclusion, the conservative management for elective surgical pathologies, could find an important place when surgery is not possible or has to be delayed, and can count, nowadays, on a wide range of medications. To get the best result, what appears crucial, is the choice of the good molecule, as not all the pain lays on the same biological base (i.e. nociceptive vs neuropathic) and making the patient aware of the choice and of the expected outcomes, in order to improve his/her compliance to the treatment, what can lead to a better result.

## References

[1] Passiatore M, Perna A, De-Vitis R, Taccardo G (2020). The Use of Alfa-Lipoic Acid-R (ALA-R) in Patients with Mild-Moderate Carpal Tunnel Syndrome: A Randomised Controlled Open Label Prospective Study.. Malays Orthop J..

[2] De Vitis R, Passiatore M, Perna A, Proietti L, Taccardo G (2020). COVID-19 contagion and contamination through hands of trauma patients: what risks and what precautions?. J Hosp Infect..

[3] Pajardi G, Bortot P, Ponti V, Novelli C (2014). Clinical usefulness of oral supplementation with alpha-lipoic Acid, curcumin phytosome, and B-group vitamins in patients with carpal tunnel syndrome undergoing surgical treatment.. Evid Based Complement Alternat Med..

[4] De Vitis R, Passiatore M, Perna A, Careri S, Cilli V, Taccardo G (2020). Seven-year clinical outcomes after collagenase injection in patients with Dupuytren’s disease: A prospective study.. J Orthop..

[5] Evangelista M, De Vitis R, Militerno A, Fanfani F (2018). Ultra-micronized Palmitoylethanolamide Effects on Sleep-wake Rhythm and Neuropathic Pain Phenotypes in Patients with Carpal Tunnel Syndrome: An Open-label, Randomized Controlled Study.. CNS Neurol Disord Drug Targets..

[6] Kucharz EJ, Szanto S, Ivanova Goycheva M, Petronijevic M, Simnovec K, Domzalski M (2019). Endorsement by Central European experts of the revised ESCEO algorithm for the management of knee osteoarthritis.. Rheumatol Int..

